# Right-Sizing Colonoscopy Surveillance Recommendations

**DOI:** 10.1016/j.gastha.2022.11.003

**Published:** 2022-11-08

**Authors:** Ashish Malhotra, Aasma Shaukat

**Affiliations:** Division of Gastroenterology, Department of Medicine, New York University Grossman School of Medicine, New York, New York

An astounding 16.6 million colonoscopies are performed annually in the United States.^1^ About 20% (3.2 million) of these examinations are surveillance procedures ^2^ largely prompted by the finding of adenomas on a previous colonoscopy. Recent improvements in colonoscopy techniques and the emphasis on adenoma detection rate presumably will increase the identification of adenomas, with an even greater need for surveillance colonoscopies in the future. This highlights the importance of conducting postpolypectomy surveillance colonoscopies at appropriate intervals not only to enhance the effectiveness of colon-cancer-prevention programs but also to minimize unnecessary procedures given finite resources and procedural risks.

In this issue of *Gastro Hep Advances*, Rosas et al ^3^ conducted a retrospective study to analyze concordance to Multisociety Task Force (MSTF) 2012 postpolypectomy surveillance colonoscopy guidelines.^4^ They analyzed 977 patients from Stanford and Palo Alto Veterans Affairs Health Care Systems divided into three time periods: preguideline (March-August 2012), postguideline (January-June 2013), and delayed postguideline (July-September 2017). Adherence to the MSTF guidelines was suboptimal and was similar in all three time periods.

The results of prior colonoscopies were not available in 22% in the preguideline and postguideline groups and in 39% in the delayed postguideline group. Furthermore, even when the report was available, pertinent information about the type and number of adenomas detected in that colonoscopy was not available in 22%–39% of cases. Authors acknowledge this challenge and suggest that increased data-sharing may help to ameliorate the issue. Although not stated in the study, the degree of lack of information between the Stanford Health System and Palo Alto VA could have been helpful to delineate the problem and possible solutions, as VA is a single-payer, single electronic health record system with good data-sharing between various VA sites.

In patients with complete polyp information from current and past colonoscopies, guideline adherence was seen only in 54%–67% of the cases. The authors acknowledge their limitation about generalizability of the study findings given the small sample size and many confounder biases such as differing endoscopist behavior (n = 48); system-level unmeasured confounders such as policies, mandates, and administrative pressures; and patient-level factors that could impact the results.

Although authors did not delve into the potential explanations for poor guideline adherence, understanding the cause is critical to develop possible solutions to address this gap. Here we have outlined few potential common reasons for the lack of adherence to guidelines:

1. Conflicting guideline recommendations
  Surprisingly, one of the most critical questions about surveillance remains unanswered. Who benefits from surveillance colonoscopy and at what frequency should it be performed? Due to the lack of these data, global variations persist between surveillance guidelines issued by different professional societies.^4^, ^5^, ^6^ For example, based on the number and size of the adenomas, the US MSTF dichotomizes patients into 2 risk categories,^4^ low- and high-risk adenomas, whereas European guidelines ^5^ propose three patient classifications, low-, intermediate-, and high-risk groups. Although each guideline is supported by a thorough literature review, paucity of controlled trial data is an impediment to the development of robust evidence-based recommendations. Trials such as Five or Ten Years (FORTE) and European Polyp Surveillance Trial (EPOS) are underway to better inform future guidelines.
2. Conflicting polyp size estimations
  Accurate assessment of the polyp diameter is a crucial step in establishing the effectiveness of a size-dependent guideline for surveillance colonoscopy. However, research using artificial colon models shows that endoscopists often underestimate or overestimate polyp size, with an overall accuracy of merely 25%–60%.^7^^,^^8^ Another notable consideration is the validity of the threshold polyp diameter of ≥1 cm, which signifies the need for aggressive surveillance. Does this recommendation have a biological reasoning? As a matter of fact, it was empirically identified from colonoscopy measurements that distinguished a group of individuals with an increased likelihood of subsequently developing advanced adenomas or carcinomas. For instance, the rate of advanced neoplasia on surveillance at 5 years was about 15% for polyps estimated to be ≥1 cm in diameter compared with 6% for 1 to 2 adenomas sized <1 cm in diameter.^9^ But studies show that polyp diameter estimation at the time of colonoscopy could be flawed.^7^^,^^8^^,^^10^ Thus, it is conceivable that present guidelines could be based on “contaminated” data in which many polyps with diameter ranging from 7 mm to 9 mm are incorrectly grouped as 10 mm or more.
3. Fear of missed cancers
  One study ^11^ showed that gastroenterologists chose a shorter surveillance interval than recommended by the guidelines due to the fears of missed cancers and malpractice concerns. Missing cancer is one of the greatest fears of endoscopists involved in screening and surveillance colonoscopies due to the perception that colon cancer is a preventable disease, and yet there is an undeniable, finite polyp miss rate.
4. Lack of familiarity with the guidelines
  Limited guideline knowledge obviously contributes to inappropriate timing of surveillance colonoscopies. Research shows that broad dissemination of guideline knowledge is challenging, and there is an estimated >10-year lag time between guideline publication and adoption.^12^ Professional societies may need to devise physician outreach and educational programs or draw from other disciplines to disseminate guideline knowledge. Electronic health record systems could also help to develop clinical workflows or decision-support systems to facilitate incorporation of guidelines into clinical practice.
5. Processes and outcomes
  Although a recommendation from the endoscopist is a crucial step in adherence to appropriate surveillance intervals, a more significant clinical outcome is whether patients actually undergo surveillance examinations at suitable, guideline-concordant intervals. This underscores the interplay of several factors that extend beyond the physician’s recommendations when evaluating guideline-concordant care such as communication with primary care providers, patient preferences, and competing medical comorbidities. Thus, guideline-concordant care is an important process measure. But the ultimate outcome measure, although more difficult to quantify, is whether colon cancers are prevented among those with a history of adenomas.

In summary, development and implementation of evidence-based colonoscopy surveillance guidelines is an important but underdeveloped area of practice. Along with gathering evidence supporting the new surveillance intervals, we must also find strategies for successful adoption of the guidelines.
